# Changes in Skin and Nasal Microbiome and Staphylococcal Species Following Treatment of Atopic Dermatitis with Dupilumab

**DOI:** 10.3390/microorganisms9071487

**Published:** 2021-07-13

**Authors:** Caroline Meyer Olesen, Anna Cäcilia Ingham, Simon Francis Thomsen, Maja-Lisa Clausen, Paal Skytt Andersen, Sofie Marie Edslev, Yasemin Topal Yüksel, Emma Guttman-Yassky, Tove Agner

**Affiliations:** 1 Department of Dermatology, Bispebjerg Hospital, 2400 Copenhagen, Denmark; simon.francis.thomsen.02@regionh.dk (S.F.T.); mlclausen@gmail.com (M.-L.C.); yasemin.topal.yueksel@regionh.dk (Y.T.Y.); tove.agner@regionh.dk (T.A.); 2 Department of Bacteria, Parasites and Fungi, Statens Serum Institute, 2400 Copenhagen, Denmark; ANMC@SSI.DK (A.C.I.); PSA@ssi.dk (P.S.A.); smed@ssi.dk (S.M.E.); 3 Laboratory of Inflammatory Skin Diseases, Department of Dermatology, Icahn School of Medicine at Mount Sinai, New York, NY 10029, USA; emma.guttman@mountsinai.org; 4 Laboratory for Investigative Dermatology, The Rockefeller University, New York, NY 10065, USA

**Keywords:** atopic dermatitis, skin microbiome, staphylococcus, dupilumab

## Abstract

Investigation of changes in the skin microbiome following treatment of atopic dermatitis (AD) with dupilumab may provide valuable insights into the skin microbiome as a therapeutic target. The aim of this study is to assess changes in the AD skin microbiome following treatment of AD with dupilumab (*n* = 27). E-swabs were collected from nose, lesional, and nonlesional skin before and after 16 weeks of dupilumab therapy, and the microbiome was analyzed by 16S rRNA and *tuf* gene sequencing. Data for 17 patients with milder disease receiving treatment with non-targeted therapies are also presented. The results show that both groups experienced clinical improvement (*p* < 0.001) following dupilumab therapy and that Shannon diversity increased and bacterial community structure changed. The relative abundance of the genus *Staphylococcus* (S.) and *S. aureus* decreased, while that of S. *epidermidis* and *S. hominis* increased. No significant changes were observed for patients receiving non-targeted treatments. The increases in *S. epidermidis* and *S. hominis* and the decrease in *S. aureus* correlated with clinical improvement. Furthermore, changes in *S. hominis* and *S. epidermidis* correlated inversely with *S. aureus*. In conclusion, treatment with dupilumab significantly changed the skin microbiome and decreased *S. aureus*. Our results suggest a favorable role of commensal staphylococci in AD.

## 1. Introduction

The pathogenesis of atopic dermatitis (AD) is characterized by the interplay between activation of the Th2/Th22-skewed immune response and epidermal barrier impairment [1,2]. This is paralleled by changes in the skin microbiome, with decreased bacterial diversity and increased colonization with *Staphylococcus aureus (S. aureus)*, which correlates with disease severity [3,4,5,6]. Conditions in AD skin promote *S. aureus* colonization [7,8], and *S. aureus* expresses a broad spectrum of virulence factors that aggravate the disease [9,10,11]. Commensal staphylococcal species, such as *S. epidermidis* and *S. hominis,* work in concert with host immunity to limit pathogen colonization [12,13,14,15], and were recently shown to be reduced in AD [16]. Accordingly, deficiency of commensal staphylococci may be related to the increased *S. aureus* colonization and disease severity in AD.

Dupilumab, a fully human monoclonal antibody targeting IL-4 and IL-13 by blocking IL-4Rα signaling [17], has been shown to improve the clinical disease activity, immune abnormalities, and epidermal barrier function, thereby highlighting IL-4Rα signaling as a key driver of the AD phenotype [18,19,20]. One previous study of changes in the skin microbiome and *S. aureus* colonization following dupilumab therapy showed that bacterial diversity increased and the absolute *S. aureus* abundance decreased [21]. However, the study did not assess treatment-related changes in other staphylococcal species and nasal microbiome.

Further understanding of the complex interactions within the bacterial communities and their cross-talk with host immunity is necessary to explore the potential of manipulating the skin microbiome in the management of AD. Accordingly, investigation of changes in the skin microbiome and staphylococcal species that are associated with successful responses to targeted and non-targeted treatments may lead to identification of genera and species that need to be further evaluated with regard to their link to AD. Therefore, the aim of the study was to investigate changes in the skin and nasal microbiome associated with successful response to dupilumab. By applying *tuf* gene sequencing, changes in the relative abundance of all staphylococcal species were assessed, revealing interesting new data on the potential interplay between different staphylococcal species following treatment.

## 2. Materials and Methods

### 2.1. Study Design

Patients were recruited from the Department of Dermatology, Bispebjerg Hospital, Copenhagen, from March 2018 to September 2019. Inclusion criteria comprised a diagnosis of AD according to UK criteria, age ≥ 18 years, and no pregnant or lactating women. Patients were scheduled to be evaluated at treatment initiation, after 16 weeks of systemic treatments (including dupilumab), and after four weeks of topical treatment. Data on the three most common loss-of-function mutations in the filaggrin gene (*FLG*) were collected [22]. Patients were instructed not to apply emollients within 24 h of each visit.

### 2.2. Sample Collection and Disease Severity Assessment

Swabs (e-Swab, Copan, Brescia, Italy) were collected from anterior nares and lesional and nonlesional skin from representative areas on the upper or lower extremities. Disease severity was assessed with the Eczema Area and Severity Index (EASI). Blood was collected for total serum IgE quantification.

### 2.3. DNA Extraction and Microbiome Characterization

DNA was extracted from swabs using an enzymatic prelysis step (30 min incubation at 37 °C with an enzyme solution containing 4 U lysostaphine (SAE0091), 25 U mutanolysin (sae0092), and 3 mg lysozyme (L4919) (Sigma-Aldrich, St. Louis, MO, USA); then 30 min incubation at 56 °C with 20 µL protein kinase K (RPROTKSOL-RO, Sigma-Aldrich, St. Louis, MO, USA), followed by DNA extraction on a MagNa-Pure 96 robot using a DNA and Viral NA Small Volume Kit (Roche, Mannheim, Germany).

The V3-V4 region of the 16S rRNA gene and that of the *tuf* gene were amplified in two separate PCRs (95 °C for 3 min; 25 cycles of 98 °C for 20 s, 60 °C for 15 s, 72 °C for 45 s; 72 °C for 5 min), using primers (16SrRNA: 341F: 5′- CCTACGGGNGGCWGCAG -3′; 805R: 5′- GACTACHVGGGTATCTAATC-3′; *tuf*: F: 5′- CAGAAGAAAAAGAACGTGG-3′; R: 5′- GTCCTCAACWGGCATCA-3′) with preceding heterogeneity spacers [23,24]. Amplicon libraries were constructed using nextera indexing primers (Illumina Inc., San Diego, CA, USA) (PCR program used: 95 °C for 3 min; 20 cycles of 98 °C for 20 s, 55 °C for 15 s, and 72 °C for 45 s; 72 °C for 5 min) and sequenced on a MiSeq instrument using a 600 cycle V3 kit (Illumina Inc., San Diego, CA, USA).

### 2.4. Sequence Pre-Processing

Demultiplexing of raw reads was performed by using the bcl2fastq Conversion Software (Illumina Inc., San Diego, CA, USA). Subsequently, cutadapt (version 2.3) [25] was used on 16S rRNA and *tuf* gene reads for heterogeneity spacer and primer trimming at an 8% error rate (corresponding to one mismatch per primer) in paired-end mode. Trimmed reads were subjected to quality filtering and amplicon sequence variant (ASV) inference with dada2 (version 1.12.1) [26]. The dada2 pipeline was utilized run-wise with default settings, except for truncation length. 16S rRNA gene reads were truncated at 270 (forward reads) and 210 bp (reverse reads), while *tuf* gene reads were truncated at 270 (forward reads) and 241 bp (reverse reads). We performed consensus chimera removal. In cases when a sample had a read count <5000 after quality filtering, it was re-sequenced. Taxonomic assignment of 16S rRNA gene sequence-derived ASVs was performed with dada2′s ‘assignTaxonomy’ and ‘addSpecies’ functions, using the Silva reference database and species-level training set (version 132) formatted for dada2, respectively. A staphylococcal-specific taxonomic database was used to classify *tuf* gene sequence-derived ASVs with the ‘assignTaxonomy’ function [24]. Using the R package phyloseq v, ASV count tables, taxonomic tables, and patient data were integrated [27]. Computational identification and removal of putative contaminants from the 16S rRNA gene sequencing data were performed for skin and nasal samples separately by using the decontam package and by additional manual filtering [28].

Twenty-five ASVs identified with the frequency method (threshold 0.01) were excluded from the skin data set, and 2041 ASVs were manually filtered out in addition. From the nasal data set, 29 ASVs were excluded based on the frequency method (threshold 0.05) and an additional 292 ASVs based on manual filtering.

Manual filtering concerned the following ASVs in both nasal and skin samples: ASVs classified no further than class-level, ASVs not classified as *Bacteria* (kingdom), and ASVs belonging to the phyla *Cyanobacteria*, *Plantomycetes*, *Chloroflexi*, and *Deinococcus-Thermus,* the class *Rhodothermia,* or the orders *Rhizobiales*, *Rhodobacterales*, *Oceanospirillales, Azospirillales*, and *Rhodospirillales*.

Read counts of re-sequenced samples were merged. One skin sample and one nasal sample, each with <4900 read counts after quality filtering, contaminant removal and merging, were excluded from downstream analyses. The final skin data set comprised 9364 ASVs, and the final nasal data set comprised 2240 ASVs.

### 2.5. Statistical Analysis

Statistical analyses and visualizations were performed using the statistical software R (version 4.0.1). The R package phyloseq was used for microbiota analysis and the ggplot2 package was used for visualization [27]. Alpha diversity was measured using Shannon’s diversity index through the phyloseq package on raw data. Shannon’s diversity index takes into account both the richness (number of different ASVs) and evenness (how evenly the ASVs are distributed) of the bacterial community. The Kruskal–Wallis test for unpaired samples and Wilcoxon’s signed rank test for paired samples were used to test for differences in Shannon diversity between visits in each group. To compare overall differences between groups, principal coordinate analysis (PCoA) plots were used for visualization, together with permutational multivariable analysis of variance, using distance matrices (PERMANOVA) tests based on Bray–Curtis distances for statistical analysis (function adonis in package vegan) [29]. This analysis was performed on Hellinger-transformed data. Homogeneity in within-group variations was tested for with the betadiper function (package vegan) as a prerequisite to applying PERMANOVA.

For barplots, all ASVs were merged at genus level, and counts were transformed to relative abundance. To assess staphylococcal species distribution, ASVs derived from *tuf* gene sequencing were transformed to relative abundance, and the 10 species with the highest percentage across all samples were plotted. Partitioning around medoid (PAM) clustering based on the Jensen–Shannon distance was used for grouping samples into an optimal number of clusters, pre-assessed by consensus of the gap statistic, silhouette width, and the elbow method (R packages cluster and factoextra) [30,31] (Maechler et al., 2019, Kassambara and Mundt). Temporal cluster dynamics were visualized in Sankey plots by utilizing the ggalluvial package [32] (Brunson, 2020). For each skin and nose site, differential abundance analyses were performed using DEseq2 [33] for the identification of differentially abundant genera, and for staphylococci differentially abundant species in relation to treatment groups, FLG mutation status, pre-versus-post treatment, and to identify changes in genera and species related to treatment response (i.e., change in EASI). Spearman’s correlation was calculated between the EASI score and Shannon diversity at baseline, as well as between change in the EASI score and Shannon diversity from baseline to post-treatment. Moreover, Spearman’s correlation was calculated between baseline relative abundances of staphylococcal species, their change in abundance from baseline to post-treatment, as well as their correlation with the EASI score. Benjamini–Hochberg multiple testing correction was used following all statistical tests when applicable. To assess changes in clinical parameters following treatment (i.e., EASI score, serum IgE, itch and sleep score, TEWL, and skin pH), Wilcoxon’s matched-pair signed rank sum test was used.

## 3. Results

### 3.1. Patient Characteristics and Clinical Response

Twenty-seven patients (mean age (range): 41.6 years (18–65); female: 14.8%; median EASI score (range): 16.3 (6.4–34.9)) were included in the study after initiating treatment with 300 mg dupilumab every second week. Patients additionally treated with topical treatment were instructed not to use topical treatments 7 days prior to each evaluation. The mean percentage reduction in the EASI score following dupilumab therapy was 70.0% (*p* < 0.001) (Table 1).

An additional 17 patients (mean age: 35.0 years (20–55); female: 47.1%; median baseline EASI score (range): 7.1 (0.8–29.1)) with milder disease receiving non-targeted treatments (TCS (*n* = 13), TCI (*n* = 1), methotrexate (*n* = 2) and azathioprine (*n* = 1)) were included in the study. The mean percentage reduction in EASI score following non-targeted therapy was 24.4% (*p* < 0.001) (Table 1).

### 3.2. Dupilumab Changed Bacterial Diversity and Staphylococci Abundance

**Figure 1:**
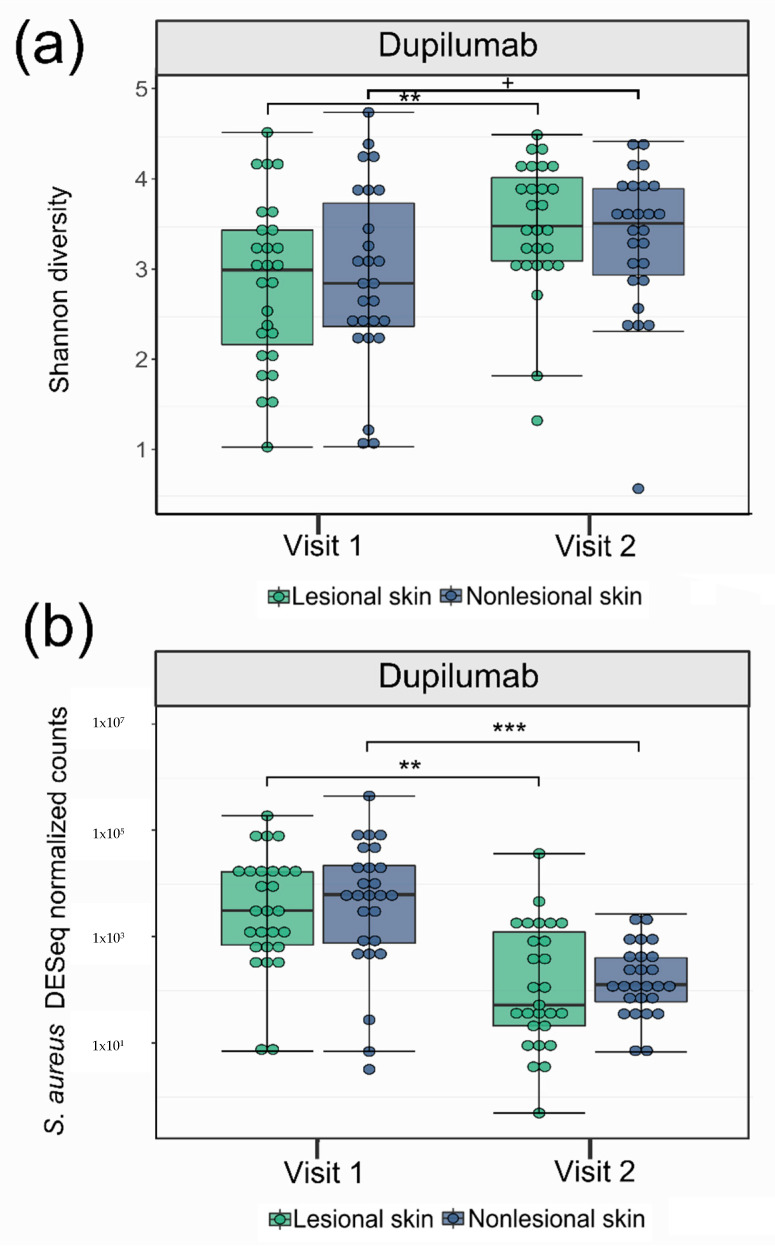
Shannon diversity and *S. aureus* relative abundance before and after treatment of atopic dermatitis patients with dupilumab. Boxplots showing (**a**) Shannon diversity and (**b**) Deseq2 normalized abundance of *S. aureus* in lesional and nonlesional skin at visit 1 and visit 2 for patients treated with dupilumab + = *p* < 0.1, ** = *p* < 0.01, *** = *p* < 0.001.

Following dupilumab therapy, Shannon diversity significantly increased on lesional skin (*p* = 0.005), and the bacterial community structure changed on both lesional and nonlesional skin (*p* < 0.001) (Figure 1a and Figure S1a,b, Table 2). The proportion of *Staphylococcus* on lesional skin (*p* = 0.02) and of *S. aureus* on lesional and nonlesional skin (*p* = 0.001, *p* < 0.001, respectively) was reduced (Figure 1b and Figure S2, Table 2), while the proportions of *S. epidermidis, S. hominis,* and *S. saprophyticus* increased on nonlesional skin (*p* < 0.001) (Figure S3a, Table 2).

Following non-targeted therapy, no significant changes in Shannon diversity, bacterial community structure, and relative abundance of genera and staphylococcal species were observed (Figures S5–S7), although the community structure of nonlesional skin showed a trend for significant change (*p* = 0.06) (Table 2).

Despite comparable post-treatment EASI scores, the bacterial community structures of lesional and nonlesional skin were significantly different between the two treatment groups, and the proportion of *S. aureus* in both lesional and nonlesional skin was significantly lower in patients treated with dupilumab (*p* < 0.001) (Table 2).

### 3.3. Shift in Composition of Bacterial Genera and Staphylococcal Species Following Treatment

Clustering on all samples revealed three clusters (Figure S4a,b). Cluster 1 was dominated by *Staphylococcus*, while cluster 2 and 3 were more diverse. Regarding staphylococcal species, cluster 1 was dominated by *S. aureus* and *S. capitis*, cluster 2 by *S. hominis* and *S. epidermidis*, and cluster 3 by a higher abundance of *S. saprophyticus* and *S. epidermidis*. The majority of patients in cluster 1 had moderate-to-severe AD (mean EASI: 17.8), while most patients in cluster 2 and 3 had mild eczema (mean EASI: 8.0 and 9.8, respectively).

Prior to dupilumab therapy, 47% of lesional and nonlesional samples were of the *S. aureus* dominated cluster 1, and the majority of these patients shifted to cluster 2 or cluster 3 following treatment (Figure S4c). A greater proportion of the patients experiencing at least 75% improvement in their EASI scores (EASI-75) shifted from cluster 1 to cluster 2 or 3. Shifts in clusters following non-targeted treatment are shown in Figure S8.

### 3.4. Correlations between the Skin Microbiome, Disease Severity and Treatment-Related Clinical Improvement

Overall, when grouping samples from the two treatment groups, bacterial Shannon diversity on lesional and nonlesional skin correlated inversely with the EASI score at baseline (*p* < 0.001), and an increase in Shannon diversity following treatment correlated with EASI improvement (*p* = 0.005 and *p* = 0.02). The proportion of *S. aureus* on nonlesional skin correlated positively with the EASI score at baseline (*p* = 0.01).

Furthermore, EASI improvement correlated significantly with a decrease in the proportion of nonlesional *S. aureus* (*p* = 0.001) and with an increased proportion of *S. hominis* on lesional and nonlesional skin (*p* = 0.003) and *S. epidermidis* on nonlesional skin (*p* = 0.003). Changes in the proportions of *S. aureus* were significantly inversely correlated with changes in *S. epidermidis* and *S. hominis* on both lesional and nonlesional skin (*p* = 0.02, *p* = 0.003 and *p* < 0.001, *p* = 0.02, respectively) (Table 3).

### 3.5. Relation between the Skin Microbiome and FLG Mutation and Serum IgE

Bacterial Shannon diversity on lesional and nonlesional skin correlated inversely with the total serum IgE at baseline (*r* = −0.29, *p* = 0.01). There was no significant difference in the skin microbiome between patients with and without FLG null mutation.

### 3.6. Characterization of the Nasal Microbiome in Relation to Treatment and Correlations with Skin Microbiome

Following dupilumab therapy, the nasal community structure changed (Table 2). Shannon diversity and community structure did not change in patients treated with non-targeted treatments.

Overall, staphylococcal species distribution did not change following treatment (Figure S3), but patients achieving EASI-75 experienced a significant increase in *S. hominis* compared to patients that did not (*p* = 0.02). However, subgroup analyses revealed that this was primarily driven by a few patients. Furthermore, relative *S. hominis* abundance in the nose correlated with the abundance of *S. hominis* on both lesional and nonlesional skin at visit 2 and on nonlesional skin at visit 1 (*p* = 0.005 and 0.02, respectively), although subgroup analysis revealed that this was likewise primarily driven by a few patients.

## 4. Discussion

In this study, we investigated changes in the skin and anterior nasal microbiome, including staphylococcal species in AD patients treated with dupilumab. Our study showed that dupilumab therapy was related to significant changes in the skin microbiome, with increased Shannon diversity and changes in bacterial community structure. Additionally, a decreased proportion of the genus *Staphylococcus* and *S. aureus* and an increase in *S. hominis* and *S. epidermidis* was observed.

Further insight into the temporal dynamics of the skin microbiome in relation to different treatments and clinical improvement is essential for exploring the potential of targeting the skin microbiome in the therapeutic management of AD [34]. At present, mechanistic evidence suggests that *S. aureus* is mutually promoted by and aggravated by Th2-mediated inflammation and skin barrier impairment in AD [10,35]. However, previous studies that evaluated the efficacy of topical antibiotics showed conflicting results [36,37], and it has been hypothesized that this may be due to recolonization by nasal *S. aureus* and eradication of beneficial bacteria [38,39]. Ongoing research is working with manipulating the skin microbiome in the therapeutic management of AD [40,41], and initial pilot trials showed that topical application of coagulase negative staphylococci (CoNS) [40] and *Roseomonas mucosa* [42] decreased disease severity.

Only one previous study has investigated changes in the skin microbiome and *S. aureus* colonization following dupilumab therapy. In line with our findings, this study demonstrated an increased Shannon diversity and a reduced absolute abundance of *S. aureus* that correlated with clinical improvement [21]. *Tuf* gene sequencing assessing the relative abundance of all staphylococcal species, not just *S. aureus*, was applied in our study. Thus, our study adds to these previous findings by elucidating the interplay between different staphylococcal species in relation to treatment, revealing a significant decrease in *S. aureus* and an increase in *S. epidermidis* and *S. hominis* proportions that correlate with clinical improvement. Furthermore, the change in proportion of *S. aureus* correlates inversely with changes in *S. epidermidis* and *S. hominis*. These findings suggest that the treatment-induced increase in CoNS might promote a sustained decrease in *S. aureus,* thereby limiting *S. aureus* re-colonization and clinical worsening of the disease. Our findings are in line with the results of previous studies demonstrating that *S. hominis* and *S. epidermidis* can produce *S. aureus*-selective antimicrobial peptides that work in concert with LL-37, a host antimicrobial peptide [15,16]. Moreover, a possible protective role of *S. hominis* in relation to the development of AD was suggested by Meylan et al. [43], showing that high *S. hominis* abundance was related to a reduced risk of developing AD in infants. Likewise, high abundance of *S. epidermidis* has been related to decreased severity of AD [4]; however, conflicting results exist regarding the role of *S. epidermidis* in AD [44].

The significant correlation between nasal and skin *S. hominis* abundance, together with a relationship between nasal *S. hominis* and clinical improvement, suggest that nasal microbiota may influence the skin microbiome by serving as a reservoir for bacteria that are transferred between nose and skin. This is supported by a previous study showing an association between nasal *S. aureus* and severity of AD [45]. The finding is interesting since it may suggest that targeting the nasal and skin microbiome simultaneously is beneficial for sustained change in the skin microbiome in some patients.

Our study showed that treatment with dupilumab was related to pronounced changes in the microbiome in both lesional and nonlesional skin and in the nose, with significant changes in bacterial community structure. Thus, our results highlight the close relationship between Th2 immune activation and global bacterial dysbiosis in AD and emphasize how blocking IL-4Rα signaling reverses the vicious circle in which *S. aureus* further promotes skin inflammation and the breakdown of the skin barrier. Interestingly, we found even stronger correlations between clinical improvement and change in relative abundance of staphylococcal species in nonlesional skin compared to lesional skin, highlighting the theoretic therapeutic potential of also including nonlesional skin in topical microbiome-based treatment strategies.

Contrary to some prior studies [5,42,43], non-targeted treatments were not related to significant changes in the skin microbiome. This may be due to the relatively few included patients, low baseline EASI scores, short follow-up time, and low relative abundance of *S. aureus* at baseline in these patients. Interestingly, despite comparable post-treatment EASI scores between the two treatment groups, patients treated with dupilumab had a significantly different community structure and lower abundance of *S. aureus* on lesional and nonlesional skin, suggesting that the impact of dupilumab on the skin microbiome extends beyond the effect of clinical improvement. A high proportion of patients treated with dupilumab reached EASI-75, supporting the promising results on the effectiveness of dupilumab.

The strength of this study is the homogenous patient cohort and the complimentary use of both 16S rRNA and *tuf* amplicon sequencing data, allowing an in-depth characterization of changes in relative abundance of staphylococcal species. Limitations include the size of the study population and lack of a control group; however, we decided to present some data from patients receiving other treatments, since treatment-related changes in general are sparsely investigated.

## 5. Conclusions

In conclusion, our study showed that targeting IL-4Rα signaling reverses the microbial dysbiosis in AD. The proportional increase in CoNS in both lesional and nonlesional skin may be particularly important for sustained treatment response by reducing *S. aureus*-induced inflammation. Furthermore, our results suggest that future microbiome-based therapeutic approaches should comprise the nasal and nonlesional, as well as the lesional, microbiome.

## Figures and Tables

**Table 1:** Demographic and clinical parameters at baseline and following treatment in the two treatment groups.

	Baseline	Follow-Up	*p*-Value ^1^
**Dupilumab treatment group ^2^**			
Age (y) mean (range)	41.6 (18–65)		
Gender, women % (*n*/ n_total_)	14.8 (4/27)		
*FLG*-mutation carrier, % (*n*/n_total_)	23.8 (5/21)		
EASI score Median (range)	(*n* = 27) 16.3 (6.4–34.9)	(*n* = 27) 3.4 (0.2–31.1)	**<0.001**
Percentage change in EASI score from baseline, % (sd)		70.0 (26.7)	
EASI-75, % (*n*/n_total_)		55.5 (15/27)	
Serum total IgE Median (min-max)	(*n* = 27) 1920 (50–23,400)	(*n* = 27) 1120 (23–12,300)	**<0.001**
**Non-targeted treatment group**			
Age (y) mean (range)	35.0 (20–55)		
Gender, women % (*n*/ n_total_)	47.1 (8/17)		
*FLG*-mutation carrier, % (*n*/n_total_)	33.3 (5/15)		
EASI score Median (min-max)	(*n* = 17) 7.1 (3.5–41.1)	(*n* = 17) 4.7 (0.8–29.0)	**<0.001**
Percentage change in EASI score from baseline, % (sd)		24.4 (53.4)	
EASI-75, % (*n*/n_total_)		23.5 (4/17)	
Serum total IgE Median (min-max)	(*n* = 15) 456 (11–20,600)	(*n* = 11) 355 (23–12,300)	0.27

^1^ Missing data were excluded for each analysis. ^2^ Abbreviations^:^ EASI; Eczema Area and Severity index, sd; standard deviation IgE; immunoglobulin E, EASI-75; proportion of patients experiencing at least a 75-percentage reduction from baseline EASI score NRS; numeric range scale (0–10 with 10 being most severe). Statistically significant *p*-values are shown in bold.

**Table 2:** Treatment related changes in microbiome and staphylococcal species in the two treatment groups ^1^.

	Dupilumab (*n* = 27)			Non-Targeted Treatment Group (*n* = 17)		
	Significant Changes Following Treatment					
	NLS	LS	Nose	NLS	LS	Nose
**Shannon Diversity**	-	+↑	+↑*	-	-	-
**Bacterial community structure**	+	+	+	-	-	-
**Bacterial genera**	-	*↓Staphylococcus*	*↑Rothia* *↑Corynebacterium* *↑Haemophilus* *↑Veillonella*	-	-	-
**staphylococcal species**	↓*S. aureus* ↑*S. epidermidis* ↑*S. hominis* ↑ *S. saprophyticus*	↓*S. aureus*	-	-	-	-

^1^ Abbreviations: LS: lesional skin; NLS; nonlesional skin; +: significant change; -: no significant change; * not significant after adjusting for multiple testing; ↑: increase; ↓: decrease.

**Table 3:** Correlations between the skin microbiome and severity, clinical improvement, and changes in *S. aureus*.

Correlations with Change in EASI Score		
**Lesional skin**	***r***	***p*-value**
Change in Shannon diversty	−0.44	**0.005**
Change in *S. aureus* relative abundance	0.29	0.06
Change in *S. hominis* relative abundance	−0.44	**0.003**
**Nonlesional skin**	***r***	***p*-value**
Change in Shannon diversity	−0.34	**0.02**
Change in *S. aureus* relative abundance	0.51	**0.001**
Change in *S. hominis* relative abundance	−0.44	**0.003**
Change in *S. epidermidis* relative abundance	−0.49	**0.002**
**Correlations with change in *S. aureus* relative abundance**		
**Lesional skin**	***r***	***p*-value**
Change in *S. hominis* relative abundance	−0.43	**0.003**
Change in *S. epidermidis* relative abundance	−0.32	**0.02**
**Nonlesional skin**	***r***	***p*-value**
Change in *S. hominis* relative abundance	−0.32	**0.02**
Change in *S. epidermidis* relative abundance	−0.54	**<0.001**

Samples from the dupilumab group (*n* = 27) and non-targeted group (*n* = 17) was merged into one group for correlation analyses. Abundance of *S. aureus* corresponds to the relative abundance (1 = 100%) in the staphylococcal species community. Correlations, *r*, were tested using spearman correlation test. Statistically significant *p*-values are shown in bold.

## Data Availability

The 16S rRNA and *tuf* sequences are available through the European Nucleotide Archive (ENA) at the European Bioinformatics Institute (EBI) under accession number PRJEB41628. Due to national data protection regulations regarding personally identifiable information, only a limited number of variables are included in the sample data file.

## Supplementary Materials

The following are available online at https://www.mdpi.com/article/10.3390/microorganisms9071487/s1, Figure S1: Changes in bacterial community structure on lesional, nonlesional, and nasal skin following dupilumab therapy, Figure S2: Relative abundance of Staphylococcus on lesional and nonlesional skin before and after dupilumab therapy, Figure S3: Changes in relative abundance of staphylococcal species following dupilumab therapy stratified by lesional, nonlesional, and nasal skin, Figure S4: Genera and staphylococci species distribution grouped in clusters and shift in clusters following treatment with dupilumab, Figure S5: Shannon diversity and S. aureus relative abundance before and after non-targeted treatment, Figure S6: Changes in bacterial community structure on lesional, nonlesional, and nasal skin following non-targeted treatment, Figure S7: Relative abundance of Staphylococcus on lesional and nonlesional skin before and after non-targeted treatment, Figure S8: Shift in clusters following non-targeted treatment.
