# Supplementary figures and images for: Changes in Skin and Nasal Microbiome and Staphylococcal Species Following Treatment of Atopic Dermatitis with Dupilumab
Source: Microorganisms. 2021 Jul 13;9(7):1487. doi: 10.3390/microorganisms9071487 (PMC8303790; doi:10.3390/microorganisms9071487)

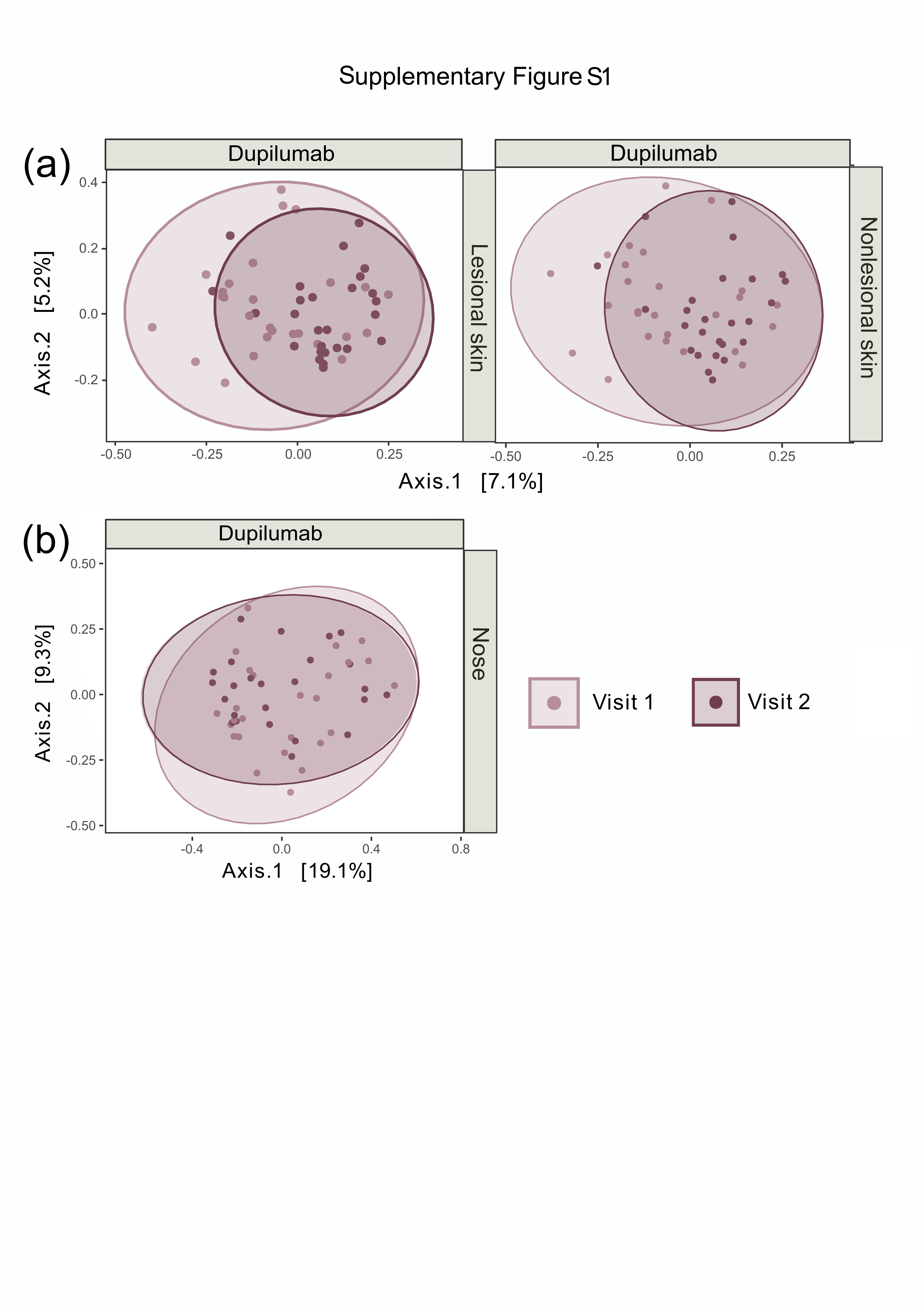

Supplement: Supplementary file 1 [file microorganisms-09-01487-s001.zip › microorganisms-1258497-SI/Supplementary figure S1.png]

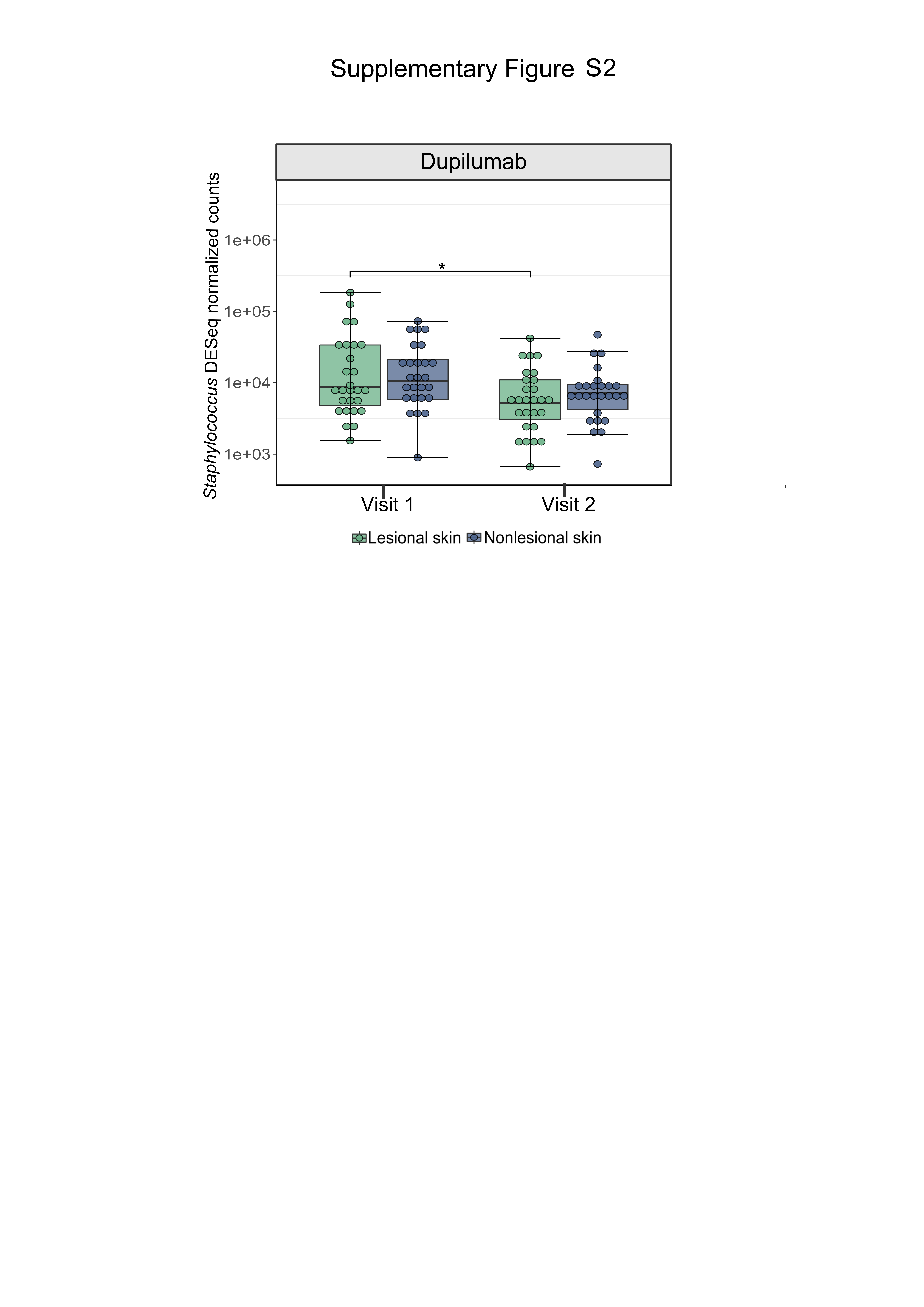

Supplement: Supplementary file 1 [file microorganisms-09-01487-s001.zip › microorganisms-1258497-SI/Supplementary figure S2.png]

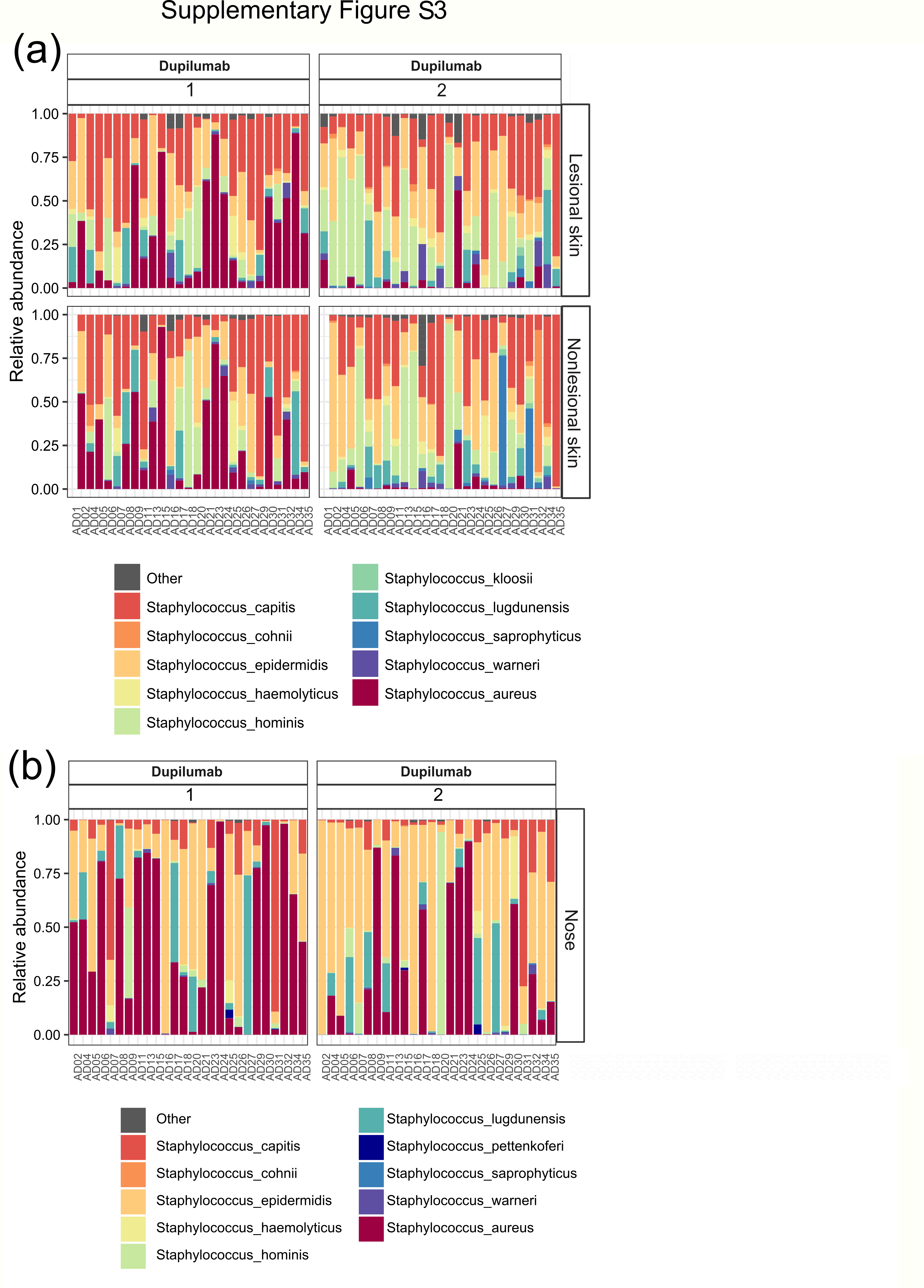

Supplement: Supplementary file 1 [file microorganisms-09-01487-s001.zip › microorganisms-1258497-SI/Supplementary figure S3.png]

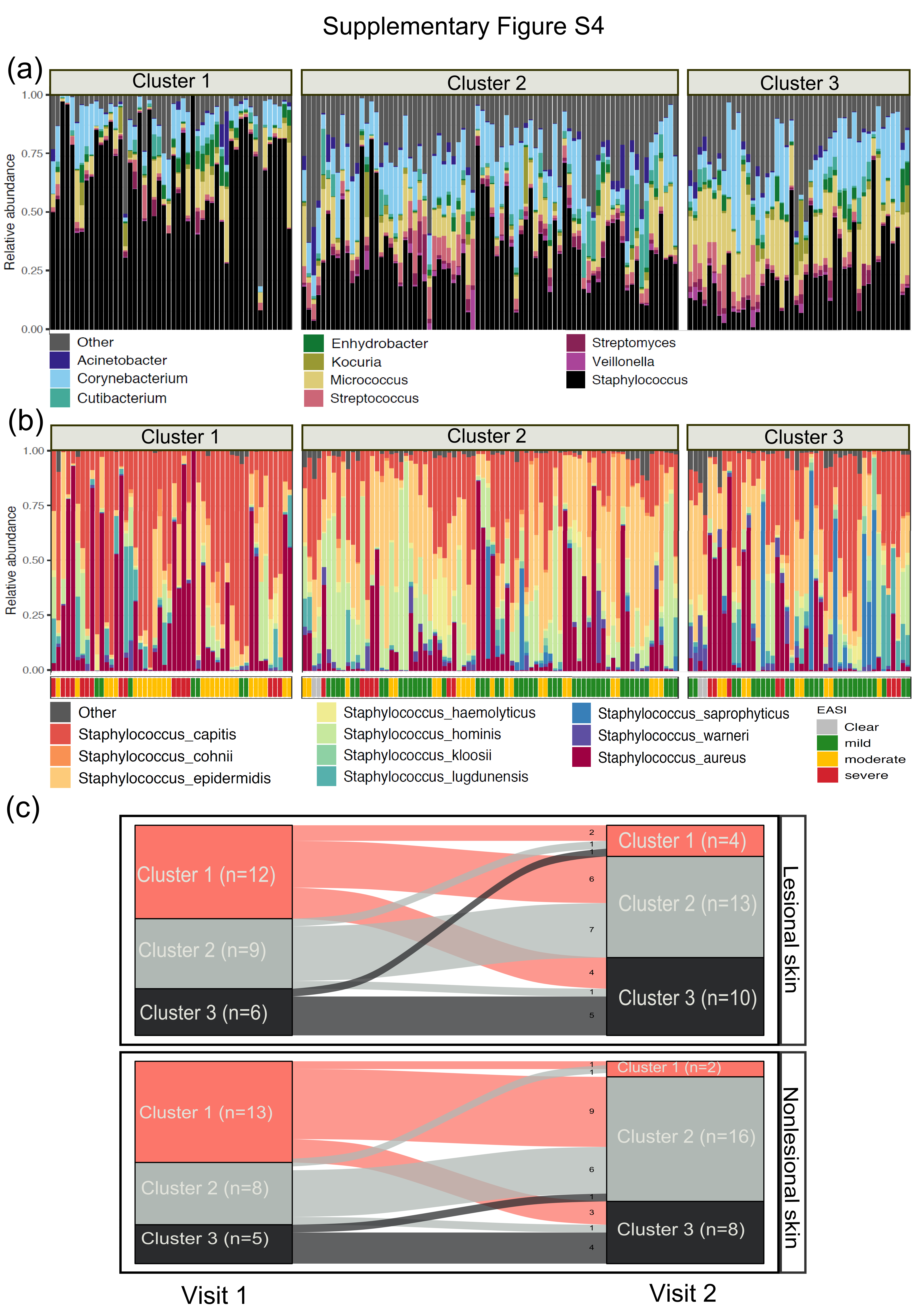

Supplement: Supplementary file 1 [file microorganisms-09-01487-s001.zip › microorganisms-1258497-SI/Supplementary figure S4.png]

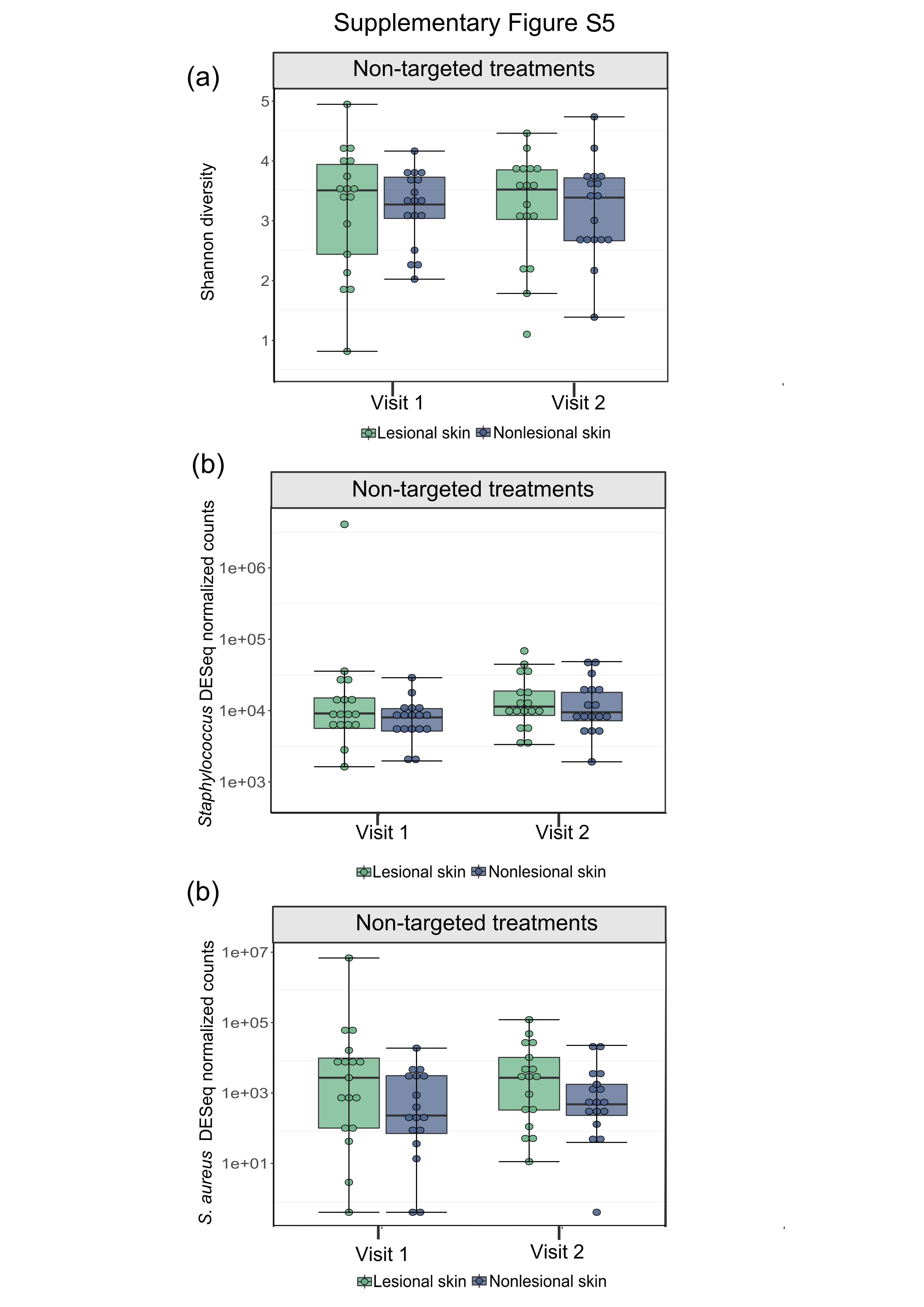

Supplement: Supplementary file 1 [file microorganisms-09-01487-s001.zip › microorganisms-1258497-SI/Supplementary figure S5.png]

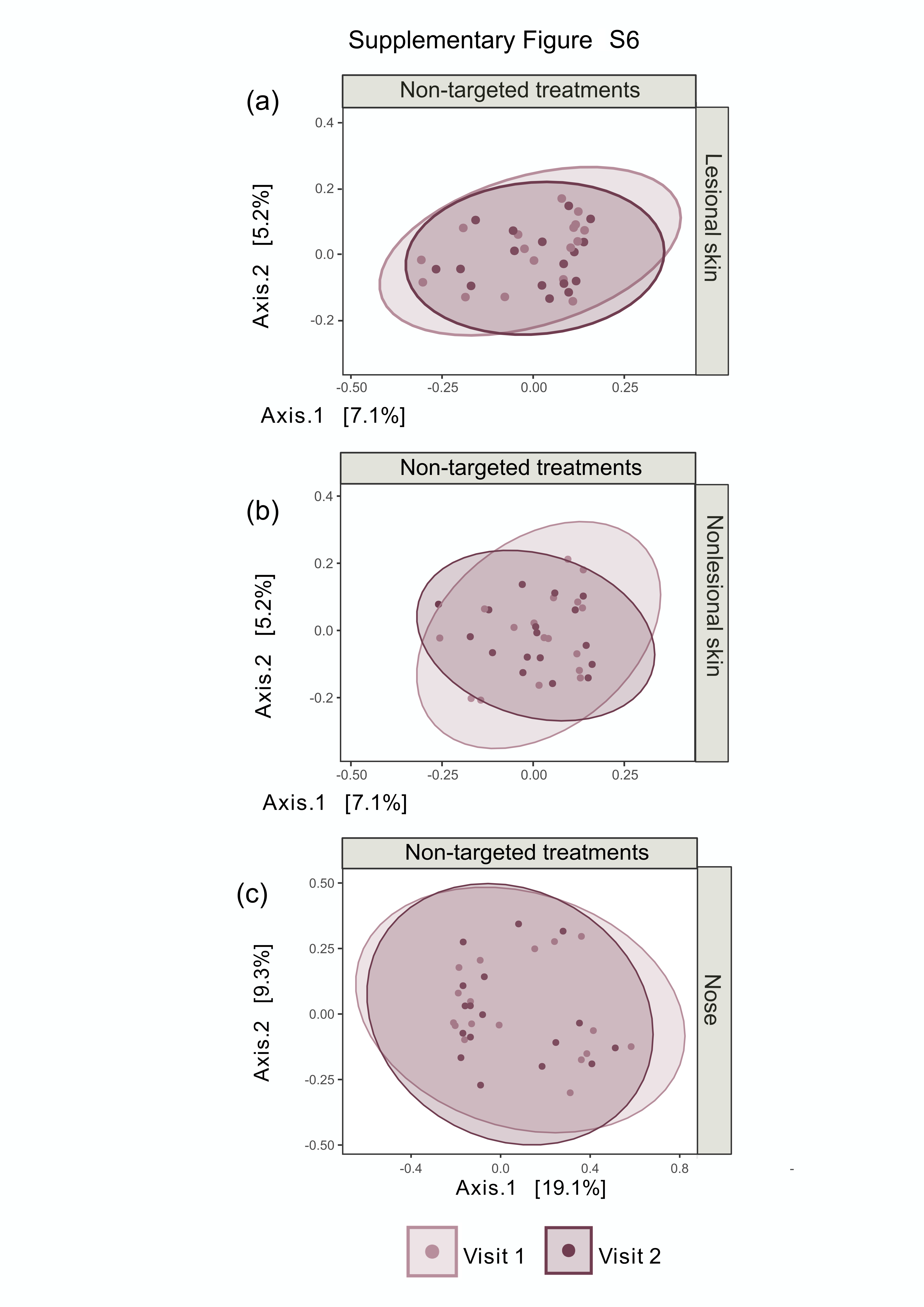

Supplement: Supplementary file 1 [file microorganisms-09-01487-s001.zip › microorganisms-1258497-SI/Supplementary figure S6.png]

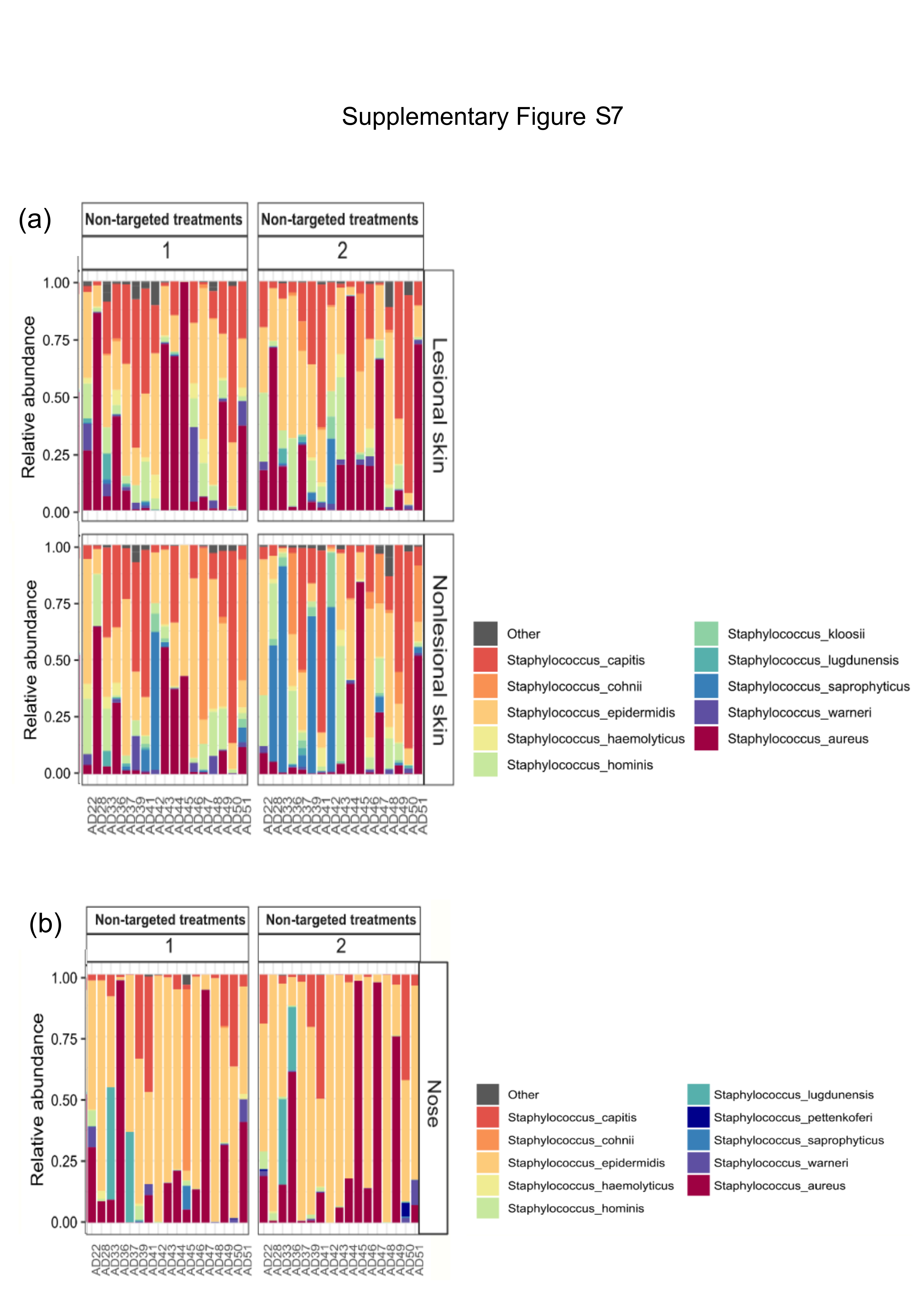

Supplement: Supplementary file 1 [file microorganisms-09-01487-s001.zip › microorganisms-1258497-SI/Supplementary figure S7.png]

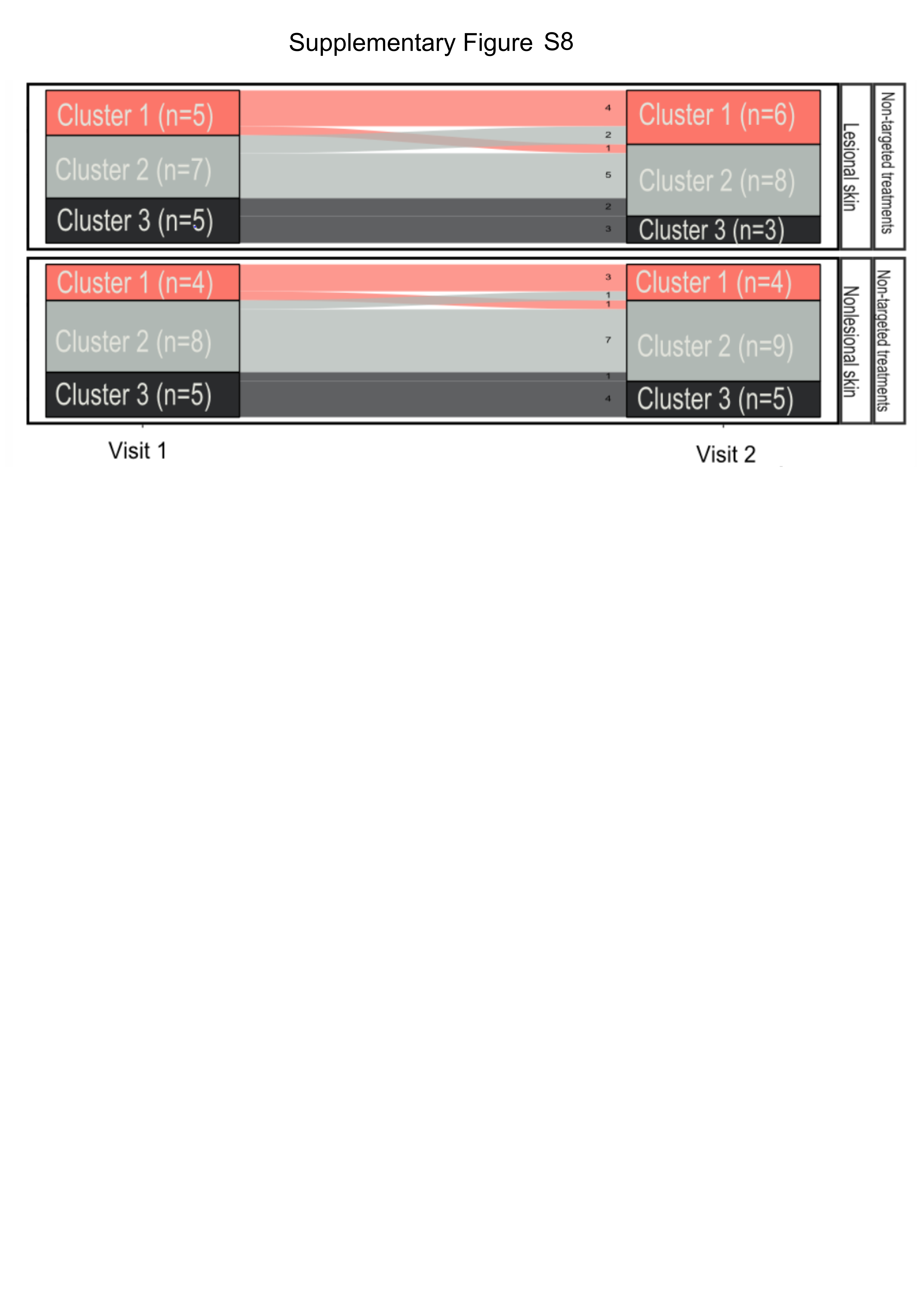

Supplement: Supplementary file 1 [file microorganisms-09-01487-s001.zip › microorganisms-1258497-SI/Supplementary figure S8.png]
